# Role of Vacancy Defects and Nitrogen Dopants for the Reduction of Oxygen on Graphene

**DOI:** 10.1021/acscatal.4c01713

**Published:** 2024-07-09

**Authors:** Weizhe Zhang, Bas van Dijk, Longfei Wu, Clément Maheu, Viorica Tudor, Jan Philipp Hofmann, Lin Jiang, Dennis Hetterscheid, Grégory F. Schneider

**Affiliations:** † Faculty of Science, Leiden Institute of Chemistry, Leiden University, Einsteinweg 55, 2333CC Leiden, The Netherlands; ‡ Department of Chemical Engineering and Chemistry, Inorganic Materials & Catalysis, Eindhoven University of Technology, Groene Loper 5, 5612AE Eindhoven, The Netherlands; § Surface Science Laboratory, Department of Materials- and Geosciences, Technical University of Darmstadt, Peter-Grünberg-Straße 4, 64287 Darmstadt, Germany; ∥ School of Microelectronics, Shanghai University, Chengzhong Road 20, 201800 Shanghai, China

**Keywords:** carbon catalyst, graphene, vacancy defect, nitrogen-doped graphite, oxygen reduction reaction

## Abstract

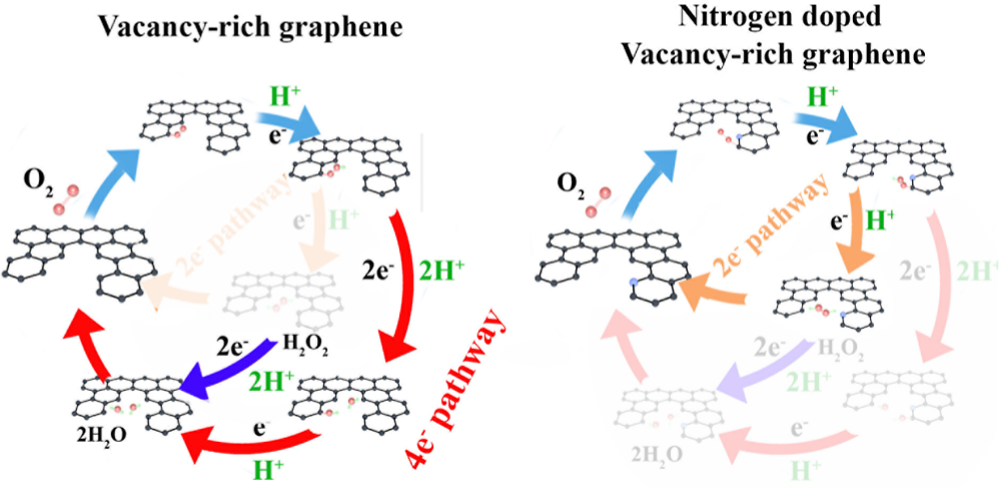

Disentangling the roles of nitrogen dopants and vacancy defects (VG) in metal-free carbon catalysts for the oxygen reduction reaction (ORR) ideally requires studying both the dopants and defects separately. Here, we systematically introduced nitrogen dopants and VGs via plasma treatment into the basal plane of monolayer graphene as a model carbon catalyst to investigate their specific roles in ORR catalysis. An increased defect density including dopants is positively associated with boosted ORR activity. Nitrogen dopants are responsible for an improved current via a 2e^–^ pathway generating hydroperoxide, while VGs result in enhanced kinetics and water production. We therefore infer that VGs in graphene are responsible for the improved ORR kinetics, while nitrogen dopants majorly influence the selectivity of ORR reaction products. The nitrogen dopants without VGs lead to a higher overpotential compared with the pristine graphene. Instead of the attribution of the ORR active site to only nitrogen species in carbon materials, the improved ORR activity in nitrogen-doped carbon materials should be attributed to the active sites constituted of VGs, oxygen dopants, and nitrogen dopants. Through this work, we provide important insights into the intertwined roles of nitrogen and VGs as well as oxygen dopants in nitrogen-doped metal-free catalysts for a more efficient ORR.

## 1 Introduction

Nitrogen-doped (N-doped) metal-free carbon catalysts (MFCCs) for the oxygen reduction reaction (ORR) have drawn considerable attention for their potentially superior activity, longer durability, and abundant storage compared to Pt catalysts.^1−11^ To realize the optimal activity of N-doped MFCCs, the identification and rational design of the active sites are required. Primarily, three chemical functions are thought to be responsible for ORR catalytic activity, with different correlations between functionalization and the catalytic process:^12−23^ (1) pyridinic N (pyri-N, nitrogen substituting one carbon of a pyridine ring at an edge) activates the adjacent carbon for ORR due to its Lewis basicity created by the electron pair donation by pyri-N.^16^ In contrast, pyri-N groups in CVD graphene (up to 16 at %) yield insufficient activity for ORR due to a large overpotential.^23^ (2) Graphitic N (grap-N, nitrogen conjugated in the sp^2^ network), however, boosts the ORR activity by significantly increasing the charge density of the adjacent pentagon carbon functioning as a graphitic carbon catalyst.^22^ (3) More generally, carbon defects, including edge and topological defects independent of N dopants, are considered as the active sites for ORR.^21^ Specifically, under acidic conditions, the pentagonal ring at the defect edge generated during plasma etching results in a higher ORR current density compared with the pyridinic sites. This is because the pentagonal ring has a higher electron-donating capability, which facilitates electron transfer to the associated oxygen.^18^ Still, the discrepancy in the ORR performance between acidic and alkaline environments can be attributed to differences in oxygen-trapping abilities. Theoretical studies have guided insights into the synthesis of carbon catalysts with controlled heteroatom-defect constitution to understand these pH-mediated differences in activity.^24^ (4) Separately, codoped with oxygen-containing groups (O dopants), pyri-N and carbon defects yield an increased ORR activity, suggesting that O dopants favor the adsorption of oxygen at the active sp^2^ carbon sites.^19^ Importantly, the increase in ORR activity in the presence of O dopants has been attributed to the enhanced kinetics because oxygen changes the local charge density^25^ and electrical conductivity compared to reduced graphene oxide (rGO) with a lower sp^2^ fraction.^26^ In addition, the selectivity of ORR is also closely related to the nature of the active sites,^26,27^ although the chemical origins and mechanisms are yet still to be understood.

MFCCs are chemically not only carbon-based, albeit often containing defects and dopants. Defects, oxygen, nitrogen, and carbon synergistically contribute to the ORR performance,^18,22,28,29^ particularly in N-doped MFCCs.^19,24^ The active site in N-doped MFCCs’ ORR performance therefore cannot be assigned directly to a single type of dopant or defect but rather by an active center being a subtle and precise combination of dopants (oxygen, nitrogen, or both) and defects.

The presence of intertwined dopants and defects in layered and thick carbon catalysts, such as graphite, hampers our understanding of the individual effects arising from defects and dopants during the initial interaction of the surface with oxygen. Graphene, consisting of a single layer of sp^2^ carbon atoms, grown by chemical vapor deposition (CVD) on copper enables the control of one factor at a time, precluding the other or to sequentially combine those. In fact, introducing oxygen in N-doped graphene showed different ORR activities than introducing nitrogen in O-doped graphene using plasma.^19^ Here, we study the particular role of vacancy defects (VG) in combination with N dopants for alkaline ORR. We employed monolayer CVD graphene, in which VGs were created using an argon plasma and further doped with nitrogen using a nitrogen plasma. In addition to VGs, graphene with vacancy-free nitrogen dopants (VF-NG) and vacancy-containing nitrogen dopants (V-NG) were prepared for a systematic comparison. Oxygen heteroatoms were confirmed to be present in all of the as-prepared samples. The catalytic kinetics and reduction pathway of doped graphene were systematically studied by evaluating the onset potential, catalytic activity, and electron transfer number. Regardless of the influence from either nitrogen dopants or VGs, the ORR activity increased with the total defect density or doping levels. Compared to pristine graphene, vacancy-free N-doping leads to a higher ORR overpotential and a selectivity toward the production of peroxide via a 2e^–^ pathway. In contrast, VGs and nitrogen doping both contribute to enhanced kinetics. The VGs contribute to a selectivity toward water production. This comparison confirms for the first time that the codoped VGs in N-doped graphene are key players in the enhancement of the ORR kinetics. Furthermore, the characterization of the chemical composition and structure–activity correlation revealed that N dopants in the vicinity of VGs contribute to increasing the ORR kinetics with a selectivity toward the 2e^–^ pathway.

## 2 Results and Discussion

### 2.1 Preparation of Doped Graphene Electrodes for ORR

**Figure 1:**
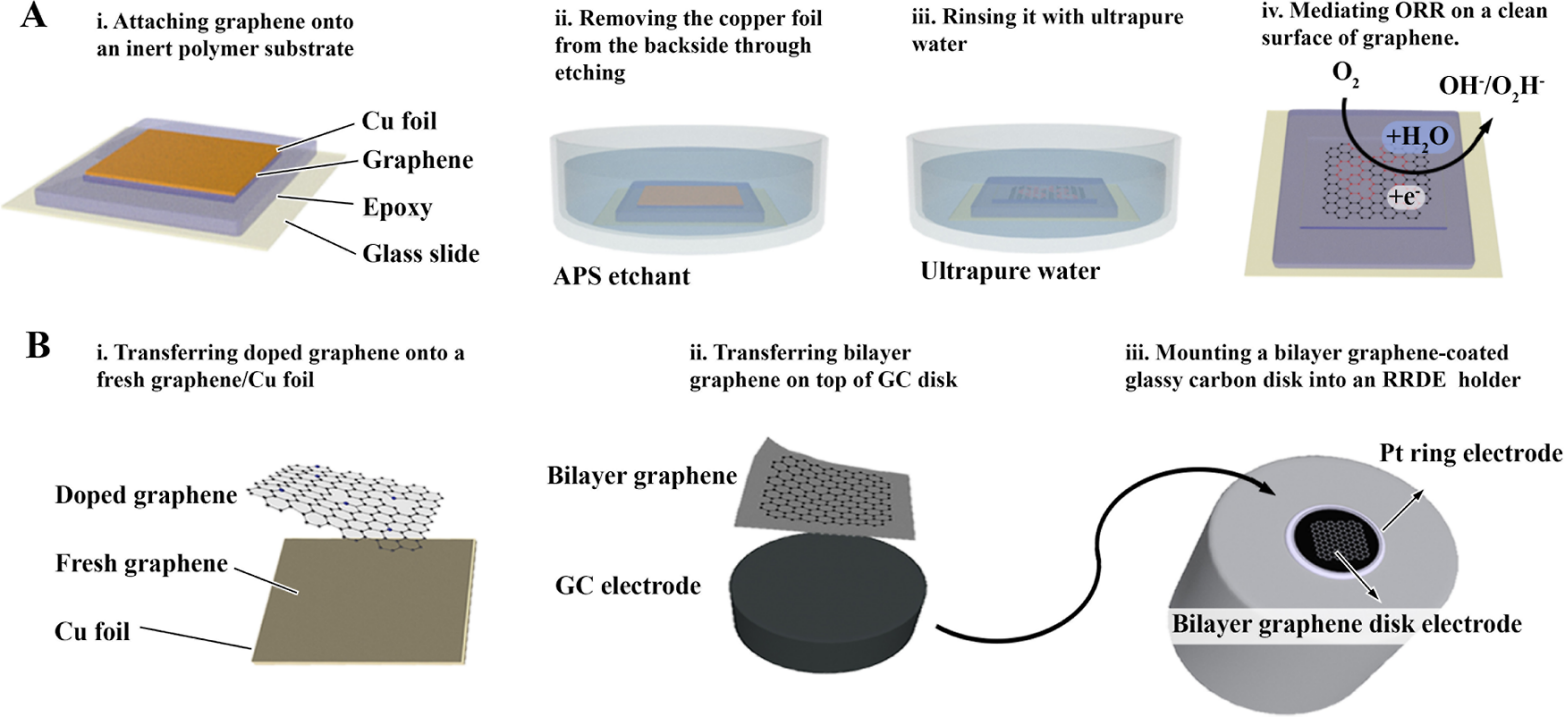
Process flow and illustration of graphene electrode preparation for ORR. (A) The first step involves fixing the graphene/Cu on a glass slide using epoxy. The Cu foil was then etched using a solution of ammonium persulfate, followed by several rinsing steps in ultrapure water. The resulting graphene, embedded in electrochemically stable epoxy, was used as a surface electrode for ORR in the polarization behavior study via linear sweep voltammetry. (B) To prepare the electrode for RRDE, bilayer graphene was transferred to a glassy carbon disk electrode. The doped graphene was transferred onto a fresh piece of graphene/Cu, forming a bilayer graphene, using a polymer-assisted transfer method. After the removal of the polymer, the bilayer graphene/glassy carbon disk electrode was mounted in the RRDE electrode holder with a platinum ring electrode. Argon and nitrogen plasma treatments.

CVD graphene was grown on a Cu film and prepared to be used as a working electrode (WE) in a three-electrode system. As depicted in Figure 1A, the side of the graphene-facing copper was prepared as an ORR electrode via an epoxy-transfer strategy.^19^ Specifically, a drop of epoxy resin was deposited on a glass slide, with the face of graphene glued to the epoxy resin and cured. Subsequently, the copper foil was etched using a 0.5 M aqueous solution of ammonium persulfate, yielding a clean graphene electrode after rinsing with a vast amount of ultrapure water (see methods).

Next, the three-electrode system was assembled by combining the graphene electrode as the WE, with a reversible hydrogen electrode (RHE) as the reference electrode (RE), and a graphite rod as the counter electrode (CE). The ORR performance was determined in a 0.1 M NaOH solution by linear sweep voltammetry (LSV).

Besides the general investigation of oxygen reduction activity via LSV, a rotating ring-disk electrode (RRDE) system was employed to investigate the ORR products (water and hydroperoxide). Figure 1B presents the process flow for the fabrication of the graphene disk electrode in the RRDE setup. For this experiment, it was essential to minimize the influence of the glassy carbon (GC) disk holder electrode. Only using a single layer of graphene did not yield a full coverage of the GC electrode. Transferring a second layer on top, however, allows covering completely the GC electrode, guaranteeing the electrochemical characterization of graphene and comparing it to its N-doped derivatives in RRDE tests (see comparison of LSV curves between bare GC and bilayer graphene-covered GC in Figure S5).^19^ To prepare this graphene bilayer electrode, a first layer of doped graphene was transferred to another pristine graphene. To do so, a layer of poly(methyl methacrylate) (PMMA) was spin-coated and cured on top of graphene on copper. The copper film was then etched away by floating the PMMA/graphene/Cu stack on a 0.5 M APS solution. The PMMA/graphene was rinsed by floating it on ultrapure water in a Petri dish, and this procedure was repeated seven times. Next, PMMA-graphene was transferred to another graphene on copper foil, creating a PMMA-bilayer graphene-Cu stack. The copper side of the graphene bilayer supported by PMMA was then floated on APS to etch the copper, resulting in a PMMA-graphene bilayer floating on APS. Finally, as-prepared bilayer CVD graphene was transferred onto the GC disk using the same transfer strategy. To remove the transferred polymer, the bilayer graphene-GC electrode was immersed in approximately 250 mL of acetone for 30 min and then rinsed with a series of organic solvents (fresh acetone, 2-isopropanol, and ethanol). This electrode is denoted as the RRDE graphene and can be compared to the graphene/epoxy/glass electrode.

**Figure 2:**
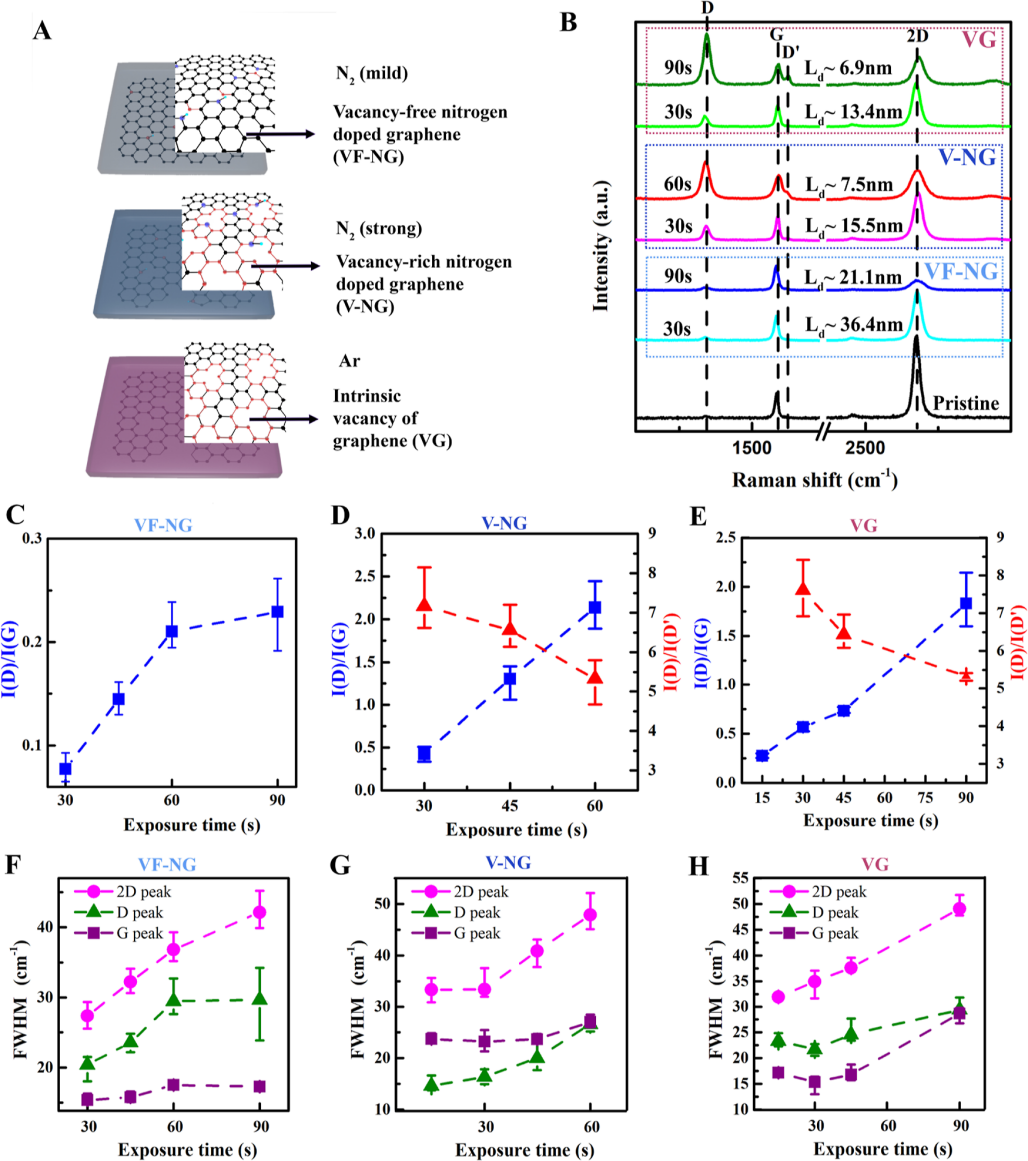
Raman spectroscopy of monolayer graphene doped with nitrogen dopants and VGs. (A) Illustration of sample preparations for vacancy-free N-doped graphene (VF-NGs) upon mild nitrogen plasma, vacancy-rich graphene (V-NGs) upon strong nitrogen plasma, and vacancy-rich graphene (VGs) without nitrogen dopants upon argon plasma. The edge of graphene is marked red (both the C=C bond and carbon atom). The purple dots are nitrogen atoms, and some of them are linked to a hydrogen atom (light blue) forming a pyrrolic nitrogen structure. (B) Raman spectra of VG, V-NG (in the dark blue square), and VF-NG (in the light blue square) with respect to the exposure time. Intensity ratios of *I*(D)/*I*(G), *I*(D)/*I*(D′) for VF-NG (C), V-NG (D), and VG (E) and fwhm’s of Raman peaks as a function of plasma exposure times for VF-NG (F), V-NG (G), and VG (H).

As shown in Figure 2A, a plasma system was employed to introduce VGs via argon plasma and N dopants via nitrogen plasma into the basal plane of graphene. The plasma treatment on graphene is well studied, yet with a high density of defects.^30−32^ We used the following conditions to control the introduction of defects and dopants at a relatively slow rate. With nitrogen plasma, mild (0.7 mbar/10 W) and strong (0.7 mbar/16 W) conditions were used, respectively, to fabricate vacancy-free N-doped graphene (VF-NG) and N-doped graphene containing vacancy defects (V-NG). During nitrogen plasma irradiation, nitrogen radicals and ions react with the graphene lattice by introducing nitrogen species. During the argon plasma irradiation (16 W, 0.4 mbar), the carbon atoms of the graphene lattice on the Cu film could be removed to prepare graphene with VG, and the defect density was tuned by using different plasma treatment times. The edges of VGs, consisting of zigzag edges, armchair edges, and Stone–Wales defects with adjacent carbon atoms,^33,34^ are considered as potential active sites for ORR due to their unique bonding states.^29,35,36^ The plasma etching procedure commonly introduces a mixture of vacancy types.^37^

Raman spectroscopy was employed to study the structure modification and homogeneity of graphene upon plasma treatment. This technique provides comprehensive information at the resolution of the laser spot (100 nm in our setup) and ensures statistical accuracy for analyzing the structure and homogeneity. Prior to Raman characterizations and after the plasma treatment, CVD graphene was first transferred onto the surface of a SiO_2_/Si wafer using PMMA-assisted transfer, as mentioned in the RRDE electrode fabrication. Figure 2B shows the Raman spectra of pristine graphene, VF-NG, V-NG, and VG evolving with plasma treatment times ranging from 30 to 90 s. Pristine graphene features two peaks: the G peak (∼1580 cm^–1^) and the 2D peak (∼2670 cm^–1^). The intensity ratio of the 2D peak (∼2670 cm^–1^) over the G peak reflects the number of graphene layers (>2 for the monolayer graphene).^38^ The introduction of defects and dopants by argon and nitrogen plasma into the graphene lattice can activate the breathing mode of the six-atom ring and induce the D peak at ∼1340 cm^–1^ and D′ peak at ∼1620 cm^–1^. In specific, the intensity ratio of *I*(D)/*I*(G) is an indicator of the defect density that can be presented as the average interdistance (*L*_d_) between two adjacent defect sites (*L*_d_ = ), where A is 102 nm^2^), while the *I*(D)/*I*(D′) ratio reflects the nature of defects and dopants.^39^ Of note, a lower *L*_d_ represents a higher defect density. The *I*(D)/*I*(G) ratio increases from 0.08 to 0.23 (*L*_d_: 36.4–21.1 nm) for VF-NGs (30–90 s mild nitrogen plasma), from 0.42 to 2.14 (*L*_d_: 15.5–7.4 nm) for V-NGs (30–60 s strong nitrogen plasma), and from 0.28 to 1.83 (*L*_d_: 27.3–6.9 nm) for VGs (15–90 s argon plasma) (Figure 2B). The homogeneity of the treated graphene lattice is indicated by the correlation between defect density and treatment time, as observed from 40 to 60 sampling points for each sample.

The differences in defect density of the above-mentioned samples indicate the different doping behaviors of the introduced dopant or defects. In particular, VF-NG shows a monotonical growth of *I*(D)/*I*(G) ratios (from 0 to 0.21 for 0 to 60 s) prior to the saturation (from 0.21 to 0.23 for 60 to 90 s) upon plasma treatments, indicating the introduction of nitrogen dopants as the defective sites in the carbon lattice (Figure 2C, top panel; Figure S1A). The absence of the D′ peak in VF-NG indicates the minimized interruption of the lattice by the dopants. In contrast, the *I*(D)/*I*(G) ratio increases from ∼0.42 to ∼2.14 for V-NGs (Figure 2D) and from 0.57 to 1.83 for VG (Figure 2E) after 30–90 s of plasma treatment. It is of note that the maximal ratio for VF-NG (∼0.23) is only 10.7–12% of that for V-NG (∼2.14) and VG (∼1.83). The significantly lower *I*(D)/*I*(G) ratio for VF-NG compared to that for V-NG and VG, together with the absence of the D′ peak in VF-NG samples, indicates the minimized interruption of the lattice by the dopants. Separately, the full width at half maximums (FWHMs) of 2D, G, and D peaks which are sensitive to the strain and structural changes^40^ are reported in Figure 2F–H. The broadening of all the Raman peaks for graphene samples upon plasma treatments confirms the systematic introduction of defects and dopants. For VF-NG, the fwhm’s increase and then saturate with the plasma treatment times (Figure 2F), showing a consistent trend with the defect density growth in Figure 2C. Similarly, fwhm’s for V-NG (Figure 2G) and for VG (Figure 2H) increase monotonically with prolonged plasma treatment time, which coincides with an increasing defect density (Figure 2D,E). Moreover, the intensity ratio of *I*(D)/*I*(D′) for V-NGs (Figure 2D) and VGs (Figure 2E) decreases from ∼7 to ∼5 for exposure times ranging from 30 to 90 s, which confirms the formation of VGs.^39^ Of note, extended plasma treatment might cause adjacent defect sites to merge, thus leading to a less accurate estimation of the defect density. Therefore, we will discuss only samples treated less than 90 s where the *I*(D)/*I*(G) increases linearly with the treatment time.

### 2.2 X-ray Photoelectron Spectroscopy Characterizations

**Figure 3:**
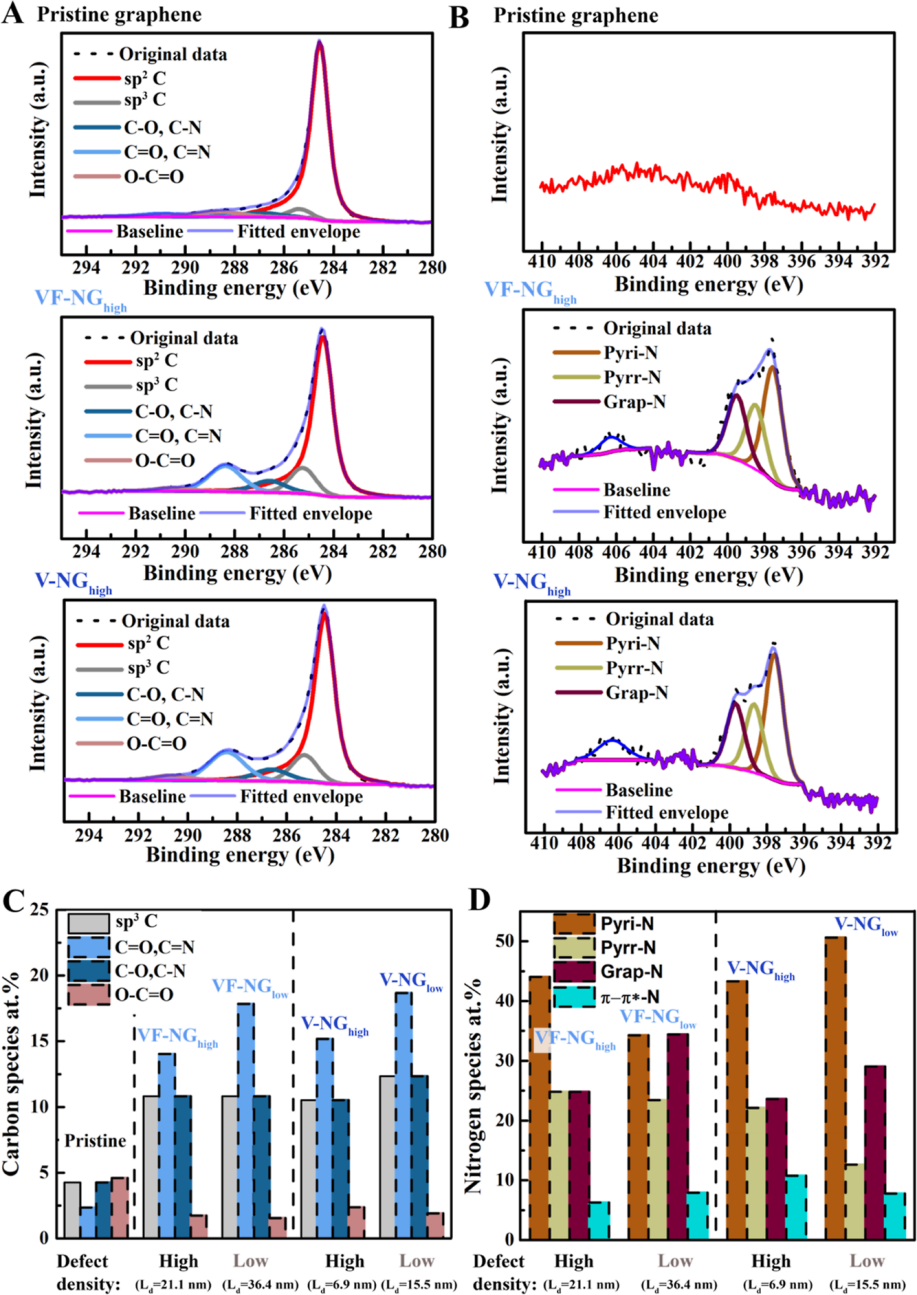
Analysis of chemical composition derived from XPS spectra for nitrogen plasma-treated graphene. (A) C 1s XPS spectra of pristine graphene, vacancy-free N-doped graphene (VF-NG_high_, *L*_d_ = 21.1 nm), and vacancy-rich graphene (V-NG_high_, *L*_d_ = 6.9 nm). (B) N 1s XPS spectra of VF-NG_high_ and V-NG_high_. (C) Constitution of carbon species (sp^3^ C, C=O/C=N, C–O/C–N, and O–C=O) for VF-NG and NG with different defect densities (indicated by the distance of two defects, *L*_d_). (D) Composition of pyri-N, pyrr-N, and grap-N derived from N 1s spectrum. LSV of VF-NG, V-NG, and VG.

X-ray photoelectron spectroscopy (XPS) was employed to characterize the chemical structure and composition of pristine graphene and the as-prepared N-doped graphene samples, namely, VF-NG and V-NG. For that, CVD graphene on copper was functionalized directly using plasma without using polymers for transfer to minimize the impact of surface contaminations induced by polymer transfer which would contribute to the XPS signal. As shown in Figure 3A, the C 1s core-level spectra of all the graphene samples can be deconvoluted into five peaks, including sp^2^ C (∼284.5 eV), sp^3^ C (∼285.3 eV), C–O/C–N (∼286.6 eV), C=O/C=N (∼288.4 eV), and O–C=O (∼290.4 eV) in Figure 3A (Figure S3a–e).^41−43^ The N 1s spectra in Figure 3B can be decomposed into four peaks: pyridinic N (pyri-N, 397.5 eV), pyrrolic N (pyrr-N, 398.6 eV), graphitic (grap-N, 400.1 eV), and π–π*–N satellite (406.5 eV) (Figure S2a-e).^44^ The satellite peak in N 1s spectra can be ascribed to higher bonding energy in π-back bonding from π* orbitals which are commonly seen in materials rich with π bonds.^45^ The N 1s spectra indicate that nitrogen plasma successfully introduces nitrogen species into pristine graphene containing no intrinsic nitrogen species.

Table 1 summarizes the compositions and species ratios derived from the C 1s and N 1s spectra of VF-NGs (with a defect density of *L*_d_ = 36.4 and 21.1 nm, referred to as VF-NG_low_ and VF-NG_high_) and V-NGs (with a defect density of *L*_d_ = 15.5 and 6.9 nm, referred to as V-NG_low_ and V-NG_high_). Due to the similar binding energy between C=O and C=N (∼288 eV) as well as C–O and C–N (∼286.5 eV) components, the isolation of C–O/C–N and C=O/C=N in C 1s spectra was performed based on the corresponding subcomponents in N 1s. The atomic ratio between nitrogen (N %) and carbon (C %) (N/C) directly indicates the nitrogen doping level. The zero N/C ratios for pristine graphene clarify the absence of nitrogen species in graphene prior to the plasma treatment. In comparison with pristine graphene, the N/C ratios of graphene samples with initial nitrogen plasma treatments increase to 0.07–0.09. Upon continuous plasma treatment, the N/C ratios decrease to 0.05 despite the growth of defect density. In detail, the N/C ratio decreases from 0.07 for VF-NG_low_ to 0.05 for VF-NG_high_ and from 0.09 for V-NG_low_ to 0.05 for V-NG_high_. Such a negative correlation between nitrogen-doping levels (N/C ratios) and defect density (*L*_d_) is unexpected. Nitrogen dopants are no longer introduced beyond a specific amount, which most likely indicates the existence of a saturation limitation.

**Table 1:** XPS Analysis of Graphene after Nitrogen Plasma^a^

		bonding composition (%)										
		C 1s (eV)							N 1s (eV)			
		284.5	285.3	286.6		288.4		290.4	397.5	398.6	400.1	406.5
samples	N/C	sp^2^ C	sp^3^ C	C–O	C–N	C=O	C=N	O–C=O	Pyri	Pyrr	Grap	Sate
pristine graphene	0.00	85.1	4.3	3.7		2.3		4.6				
VF-NG_low_	0.07	62.7	10.8	3.8	2.7	11.8	4.6	1.4	2.7	2.7	1.9	0.6
VF-NG_high_	**0.05**	67.1	10.8	5.0	1.1	10.5	2.9	1.7	1.9	1.1	1.1	0.3
V-NG_low_	0.09	60.2	12.4	3.8	2.5	11.6	5.5	1.7	4.4	2.5	1.1	0.7
V-NG_high_	**0.05**	65.6	10.5	5.0	1.1	11.5	3.0	2.3	2.0	1.1	1.0	0.5

a The ratio of nitrogen to carbon (N/C) and composition of chemical bonds in nitrogenated graphene from XPS results.

According to the spectra in Figure 3A,B, the composition of the carbon and nitrogen species in VF-NGs and V-NGs was derived in Figure 3C,D. Compared with pristine graphene, both the VF-NG and V-NG samples exhibit decreased sp^2^ C ratios and increased sp^3^ C ratios, confirming the generation of defects in graphene upon nitrogen plasma treatment. However, upon an increase in the defect density, VF-NG_high_ exhibits an increased sp^2^ C ratio (67.1%) and a retained sp^3^ C ratio (10.8%) compared to VF-NG_low_ (sp^2^ C: 62.7%, sp^3^ C: 10.8%). Similarly, V-NG_high_ exhibits an increased sp^2^ C ratio (65.6%) and sp^3^ C ratio (12.4%) compared to V-NG_low_ (sp^2^ C: 60.2%, sp^3^ C: 10.5%). Meanwhile, the C–N/C=N ratios decrease with the defect density. The C–N component ratio decreases from 2.7% for VF-NG_low_ to 1.1%, for example. These results coincide with the changing trend of the N/C ratios upon nitrogen doping and can be ascribed to the absence and presence of VGs in the two types of N-doped samples. As discussed above, the decreased nitrogen content in comparison with the increased defect density upon increased plasma treatment times can be rationalized by the growth of components excluding C and N, namely, the O components. The analysis of the O components is determined to be based on the C 1s peaks instead of the O 1s ones to exclude the impurities from copper oxides originating from the as-grown substrate of graphene. As seen from Table 1, C–O and O–C=O ratios increase from 3.8 and 1.4% for VF-NG_low_ to 5.0 and 1.7% for VF-NG_high_. As a consequence, the carbon–oxygen contents of the graphene samples also evolve with the nitrogen plasma treatment, which is expected to contribute to the ORR catalysis.^19^ In addition, the lower O–C=O ratios (∼1.4 to 2.3%) in N-doped graphene compared to pristine graphene (4.6%) could be ascribed to the removal of surface contaminations on graphene by the plasma treatment, which also indicates the presence of surface impurities in pristine graphene (i.e., graphene facing the air on the copper foil).

In particular, the VF-NG_high_ and V-NG_high_ samples have the same N/C ratio values (0.05) and similar chemical composition. The only differences between the two samples are the defect nature and defect density reflected by Raman spectroscopy. Meanwhile, V-NG_high_ and VG_high_ with the *L*_d_ of 6.9 nm and *L*_d_ of 7.5 nm have similar levels of defect density (Figure 2). Therefore, the highly similar chemical components in VF-NG_high_ and V-NG_high_, as well as the similar levels of defect density between V-NG_high_ and VG_high_, allow us to investigate the individual and synergistic roles of nitrogen dopants and VGs.

**Figure 4:**
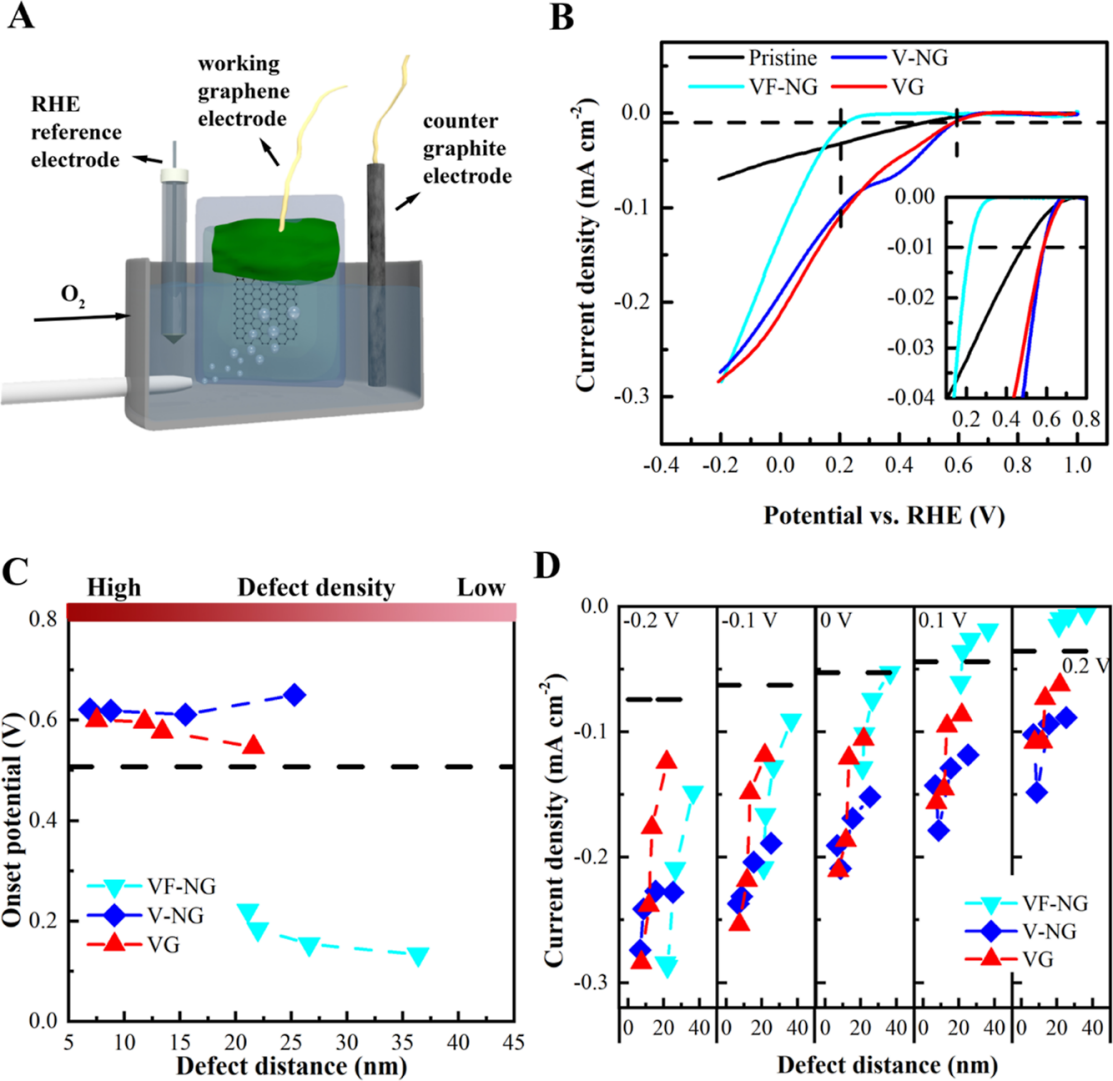
ORR polarization curves (LSV) and ORR activity of argon and nitrogen plasma-treated graphene monolayer. (A) Illustration of the electrochemical cell containing the graphene electrode, a counter graphite electrode, and a reversible hydrogen RE. Measurements were conducted in an oxygen-saturated 0.1 M NaOH solution at 100 mV/s scan rate. (B) LSV curves of pristine graphene, VF-NG (*L*_d_ = 21.1 nm), V-NG (*L*_d_ = 6.9 nm), and VG (*L*_d_ = 7.5 nm). The dashed line indicates the defined onset potential (*E*_onset_) at a current density of 0.01 mA cm^–2^. Inset: zoom-in region of onset potential. (C) *E*_onset_ for VF-NG, V-NG, and VG vs defect distance *L*_d_. (D) Current density for NG, V-NG, and VG at the voltage of −0.2, −0.1, 0, 0.1, and 0.2 V, obtained from the LSV curves, vs the corresponding defect distance *L*_d_. The dashed lines indicate the current densities of pristine graphene under specific voltages.

The electrochemical activity of the ORR for graphene before and after doping with nitrogen species and/or VGs was studied using LSV. The ORR activity in alkaline media (0.1 M NaOH) shows higher activity compared to measurements in acidic media (0.1 M H_2_SO_4_), as demonstrated in our previous study.^19^ To compare the ORR performance of our as-prepared graphene electrodes under conditions showing a higher ORR activity, we conducted all measurements in 0.1 M NaOH. Figure 4A shows the electrochemical measurement setup, including graphene as the WE. Figure 4B presents the LSV curves of VF-NG, V-NG, and VG with the highest defect density (VF-NG with *L*_d_ = 21.1 nm, V-NG with *L*_d_ = 6.9 nm, and VG with *L*_d_ = 7.5 nm). Though the defect density of VF-NG is much lower than that of V-NG and VG, it has a similar amount of N dopants compared to that of V-NG, which makes the comparison between VF-NG and V-NG distinguishable for the role of VGs. The onset potential (*E*_onset_ versus RHE, marked with a dashed line for the three samples in Figure 4B)—defined as the potential when the current density achieves 0.01 mA cm^–2^—indicates the potential at which the ORR initiates. Compared to pristine graphene (*E*_onset_ = 0.51 V), the onset potential of VF-NG shifts negatively to 0.22 V, indicating more sluggish ORR kinetics in the case of VF-NG. In contrast, the onset potentials of V-NG and VG shift positively to 0.60–0.62 V. Such a contrast suggests the importance of VGs for improving the ORR kinetics both in N-doped and vacancy-rich graphene. Moreover, the impact of the defect density on the ORR activity for doped graphene was systematically investigated. Figure 4C summarizes the onset potentials, and Figure 4D summarizes the current density under specific voltages as a function of the defect density *L*_d_ (obtained from LSV curves presented in Figures 4B and S4). In general, *E*_onset_ demonstrates a positive correlation with the defect density; a more positive *E*_onset_ is associated with a higher defect density (lower *L*_d_ values) for VF-NGs and VGs. *E*_onset_ first decreases with the increase in defect density for V-NGs, which is most likely due to the decrease in nitrogen dopants during the increase in doping levels based on the previous XPS results.

Furthermore, the current densities of the graphene electrodes increase when the defect density increases. Figure 4D compares the increasing trends of the current densities as a function of the defect density under a series of applied voltages (0.2 to −0.2 V). The current densities increase with the negative shift of applied voltages, in which V-NG generally shows superior current levels compared to VF-NG and VG. The current densities rise to a similar level of −0.28 mA cm^–2^ for VF-NG (*L*_d_ = 21.1 nm), V-NG (*L*_d_ = 6.9 nm), and VG (*L*_d_ = 7.5 nm), which are ∼4 times higher than the pristine graphene (−0.07 mA cm^–2^, indicated by the dashed line in Figure 4D). At an applied voltage of 0.2 V, VF-NG (0.005 mA cm^–2^ for *L*_d_ = 36.4 nm) has much lower current densities compared to V-NG (−0.09 mA cm^–2^ for *L*_d_ = 25.3 nm) and VG (−0.06 mA cm^–2^ for *L*_d_ = 21.6 nm). For the highest defect density, V-NG and VG have higher current densities of −0.11 mA cm^–2^ compared to VF-NG (−0.02 mA cm^–2^). Regarding the change of chemical components, discussed in the previous section, the increased defect density leads to a decrease in N % and an increase in O % and sp^2^ C in both VF-NG and V-NG. It is therefore assumed that sp^2^ C and O contents accompanying the N dopants in N-doped graphene samples possibly favor the enhanced ORR activity.

### 2.3 Rotating Ring-Disk Electrode Characterization for VF-NG, V-NG, and VG

**Figure 5:**
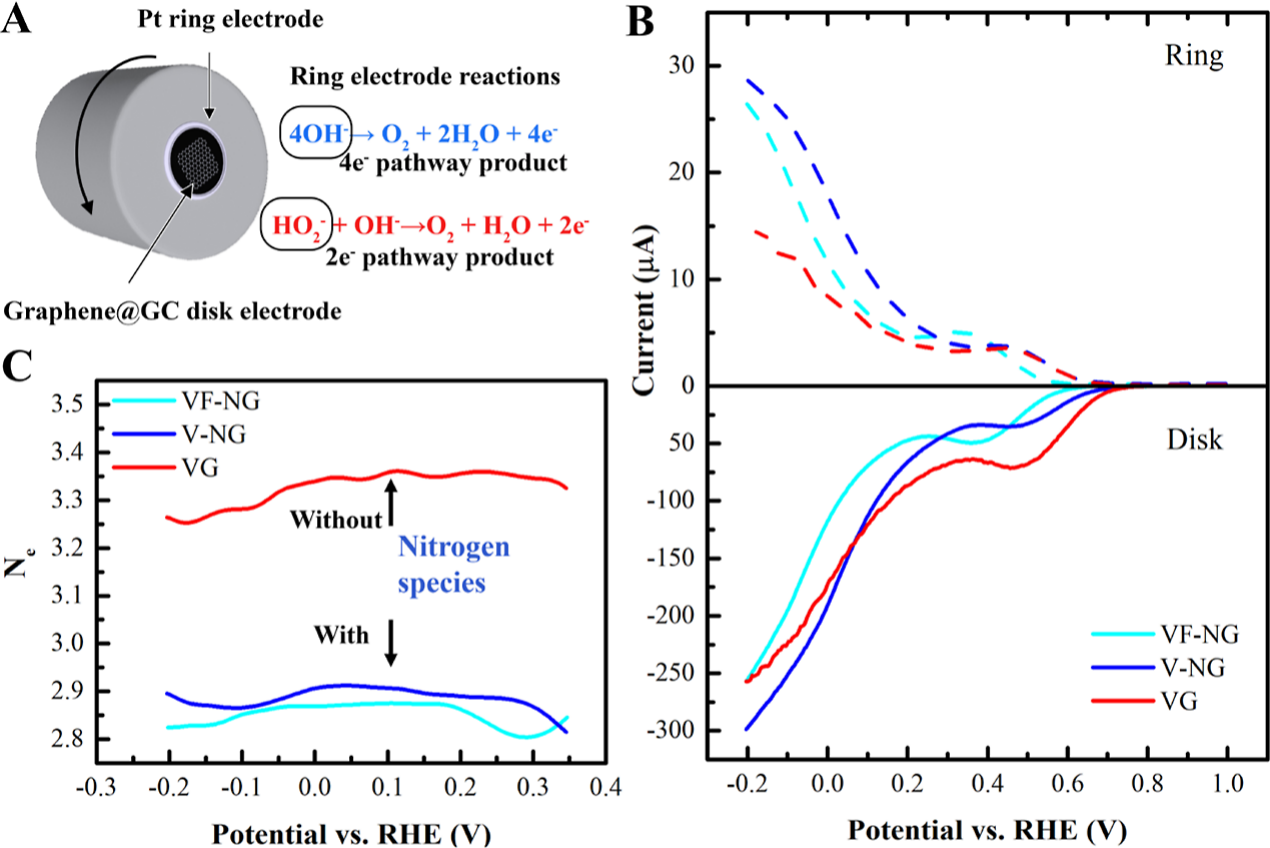
RRDE measurements of VF-NG, V-NG, and VG on the disk electrode of GC. (A) Illustration showing the RRDE electrode preparation. (B) LSV curves (including disk and ring currents) at a rotation speed of 800 rpm for VG, V-NG, and VF-NG. (C) Electron transfer numbers (*N*_e_) for VG, VF-NG, and V-NG as a function of applied potential. Measurements were conducted in an oxygen-saturated 0.1 M NaOH solution at 100 mV/s scan rate.

A rotating ring-disk electrode (RRDE) system was applied to investigate the ORR selectivity of graphene in a 0.1 M NaOH solution with a graphite rod as the CE and an RHE as the RE. Notably, determining the electron transfer number using RRDE requires a stable WE (i.e., graphene@GC) and accurate estimation of the products oxidized at the ring electrode. We found no change in the Raman spectra of the GC electrode after the RRDE measurements, as reported in our previous work, and the collection efficiency is 22.5%.^19^ The RRDE graphene (bilayer graphene on the GC disk, illustrated in Section 1) reduces the oxygen, and the ORR products (i.e., H_2_O_2_) are oxidized by a platinum ring (Figure 5A). The comparison between the ring and disk currents allows us to distinguish the reaction selectivity toward the 4e^–^ pathway (H_2_O as product) and the 2e^–^ pathway (HO_2_^–^ as product). The oxygen reduction occurs in alkaline conditions as follows
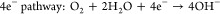

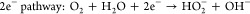


Figure 5B shows the RRDE LSV curves at ring and disk electrodes of VF-NG, V-NG, and VG (VF-NG with *L*_d_ = 21.1 nm, V-NG with *L*_d_ = 6.9 nm, and VG with *L*_d_ = 7.5 nm, the same to samples in Figure 3B for the ease of comparison). During the initial RRDE measurement, the rotation speed was varied between 400 and 2500 rpm (Figure S6). We selected 800 rpm for the following discussion to avoid any disturbance, most likely owing to the delamination or damage of graphene, in LSV curves with high rotation speeds (>1000 rpm).

The electron transfer number *N*_e_ is calculated based on the following equation^46^
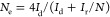
1where *N* is the current collection efficiency of the ring electrode, *I*_d_ is the disk electrode (RRDE graphene) current, and *I*_r_ is the Pt ring current obtained from the oxidation of products diffused from the disk. Figure 5C further summarizes the *N*_e_ in the potential range of −0.2 to 0.2 V. In ideal cases where only one reaction route happens, *N*_e_ is 4 for the 4e^–^ pathway and *N*_e_ is 2 for the 2e^–^ pathway. The average *N*_e_ of VF-NG under the voltage between 0.4 and −0.2 V is in the range of 2.8–2.9, which is similar to V-NG, indicating the same 2e^–^ pathway with a considerable amount of peroxide generated. In contrast, the *N*_e_ of VG falls in a range from 3.25 to 3.35, indicating that the major product is the water of ORR via either a direct 4e^–^ pathway or via a faster reduction of peroxide to water. Such a contrast in the ORR selectivity can be attributed to change in the charge states of the active carbon atoms modulated by N dopants, resulting in differences in the electrochemical barrier and preference to produce water or hydrogen peroxide.^47^ Therefore, it is concluded that nitrogen dopants in graphene could lead to a higher yield of peroxide. In comparison, vacancies in doped graphene contribute to the production of water as an ORR product.

### 2.4 General Overview of the Active Center in Carbon Catalysts for ORR

For a better understanding of ORR occurring on defect-rich and heteroatom-doped carbon catalysts, we summarize here our findings and compare those with state-of-the-art theoretical and experimental reports. Nitrogen dopants, VGs, as well as oxygen dopants in the sp^2^ basal plane carbon network have been systematically studied for their role in the ORR activity and selectivity. As identified by Raman (defect density), XPS (chemical compositions), and electrochemical characterizations (ORR activity and selectivity), the intertwined roles of the various defects can be disentangled. (1) VGs are responsible for the decreased overpotential, especially compared to N dopants without VGs. (2) Increased densities of all of the defects contribute to the improvement of ORR activity. (3) Oxygen dopants, particularly the C–O component, have shown a positive correlation with improved ORR activity. Especially for the N-doped samples, the N % decreases, while the O % increases with a prolonged treatment time. The ORR activity also increases with the prolonged treatment time. (4) N dopants increase the selectivity of the ORRs toward the two-electron path with peroxide as a product, regardless of the existence of any VG.

#### 2.4.1 Role of VGs

The overpotential originates from the energy barrier of the adsorption and desorption of oxygen. In the vacancy-rich samples, the defects with an open area near the graphene edge provide more space for the adsorption of oxygen and therefore lower the adsorption energy. A theoretical prediction about the difference in energy barrier for the adsorption of O_2_ at the edge vs basal plane showed that an edge efficiently lowers the O_2_ adsorption energy.^47^ Additionally, higher current densities were observed for N-doped graphene with VGs compared with the pristine graphene and vacancy-free N-doped graphene. In our previous work, O dopants were found to be essential for the improvement of ORR activity in N-doped and vacancy-rich graphene.^19^ Previous reports have focused on the edges, at the end of the basal plane, to understand the higher activity of the ORR at edges compared with the basal plane. It has been found that zigzag edges are active for the adsorption of oxygen.^29,48^ However, unlike the edges of a large lattice that make it easy to find a correlation with the increased ORR activity, the edges of VGs are often overlooked. This is particularly true when there is a more distinguishable factor, such as the introduction of N dopants on purpose. Thus, the actual active center in the doped carbon catalysts for ORR could be illustrated as defects adjacent to heteroatoms (O and N),^24^ instead of the active sites attributed to a single factor such as a specific dopant. To increase the density of the edges, it should not be limited to downscale the size of carbon flakes or aligning end edges to oxygen. The VGs actually provide sufficient, even more, edges in the continuum basal plane.

#### 2.4.2 Roles of Heteroatom Dopants

The addition of dopants is known to alter the selectivity of products, from water to peroxide, by modulating the electronic charge states of the active carbon atoms.^49^ Theoretical research on O_2_ adsorption on zigzag edges, with and without nitrogen dopants, indicates that the zigzag edge has the lowest energy barrier for O_2_ adsorption. In contrast, the presence of nitrogen dopants increases the O_2_ adsorption energy barrier at these edge sites.^47^ Nitrogen dopants also influence the electronic structure of the lattice, resulting in an altered O_2_ adsorption energy on the graphene lattice. Theoretical studies have found that Grap-N facilitates the ORR by introducing 1.5–2 electrons into the lattice, while Pyri-N contributes minimally to decreasing the O_2_ adsorption energy.^50^ In contrast to the VGs that led to water as the product, the N dopants led to a change in product selectivity from water to peroxide via a 2e^–^ pathway. A similar selectivity change derived from the N dopants has been reported with graphene oxide (GO, O/C > 0.1 based on XPS results).^26^ Another factor is the O dopant, which is unavoidable, owing to the oxygen adsorption to the active carbon atoms. The O dopant levels in VGs (with ∼0 2 mol/L of O/C) increase with the increase in defect density and lead to no change in the preference in the reaction route. Therefore, the O dopants have not contributed to the change in the product selectivity but do lead to an increase in the activity. Based on the confirmed influences from VGs and O dopants that will not change the water as a major ORR product, it can be concluded that the introduction of nitrogen dopants favors the formation of peroxide and allows for higher current densities, instead of a lower overpotential.

## 3 Conclusions

To demonstrate the role of dopants and defects in N-doped carbon materials for ORR, we systematically introduced nitrogen dopants and VGs in a model carbon catalyst of monolayer graphene via nitrogen and argon plasma treatments. VGs lead to a higher ORR activity with or without nitrogen dopants and especially to a decrease of the overpotential. N dopants can also improve the ORR current under a negative enough voltage (←0.1 V). While in the vacancy-less case, N dopants increase the overpotential. Moreover, N dopants lead to an increased selectivity of the 2e^–^ pathway with more peroxide products. To develop a more reactive carbon ORR catalyst, we thus need to introduce more VGs while avoiding N dopants to prevent the formation of peroxide in systems such as fuel cells. In situations where peroxide production is desired, the synergistic effects of N dopants and VGs should be maximized to achieve a high selectivity toward peroxide products while decreasing the overpotential.

## Supporting Information Available

Materials and methods for the graphene electrode preparation and electrochemical measurements; supplementary Raman and XPS spectra; and supplementary electrochemical results (PDF)
