# Supplementary material for: Role of Vacancy Defects and Nitrogen Dopants for the Reduction of Oxygen on Graphene
Source: ACS Catal. 2024 Jul 9;14(14):11065–75. doi: 10.1021/acscatal.4c01713 (PMC11264207; doi:10.1021/acscatal.4c01713)
Supplement: Supplementary file 1 — cs4c01713_si_001.pdf [file cs4c01713_si_001.pdf]

Supporting Information

for

The role of vacancy defects and nitrogen dopants for the reduction of oxygen on graphene

Weizhe Zhang[1], Bas van Dijk[1], Longfei Wu[2], Clément Maheu[3], Viorica Tudor[1], Jan Philipp Hofmann[2,3], Lin Jiang[1,4*], Dennis Hetterscheid[1*], Grégory F. Schneider[1*]

[1] *Leiden University, Faculty of Science, Leiden Institute of Chemistry, Einsteinweg 55, 2333CC Leiden, The Netherland*

[2] *Eindhoven University of Technology, Department of Chemical Engineering and Chemistry, Inorganic Materials & Catalysis, Groene Loper 5, 5612 AE Eindhoven, The Netherlands*

[3] *Technical University of Darmstadt, Department of Materials and Earth Sciences, Surface Science Laboratory, Otto-Berndt-Strasse 3, 64287 Darmstadt, Germany*

[4] *Shanghai University, School of Microelectronics, Chengzhong Road 20, 201800, Shanghai, China*

* To whom correspondence should be addressed:

g.f.schneider@chem.leidenuniv.nl

d.g.h.hetterscheid@chem.leidenuniv.nl

linjiang@shu.edu.cn

1. Materials and Methods

Materials and chemicals. Chemical vapor deposition graphene (CVDG) was grown in a commercially available tube oven (planarGROW-2B, planarTECH) on a thin foil made of copper (Alfa Aesar, 99.999% purity, 25 μm thickness) by annealing and stabilizing the copper foil at 1035 °C in an atmosphere of H_2 (g, 40 mTorr) following with introducing 35 sccm CH_4 (g, 500mTorr) to the Cu surface for the desired period. Sodium hydroxide monohydrate ($NaOH \cdot H_2O$, TraceSelect≥99.9996%) were purchased from Honeywell Fluka. The solutions were prepared with water from the Millipore Milli-Q gradient A10 system (18.2 MΩ cm). Poly(methyl methacrylate) (PMMA) was purchased from All resist GmbH (6% in anisole, AR-P 662.06), and Epoxy resin along with curing agent was purchased from GENTEC.

Plasma treatment. We conducted the plasma treatment using a capacitively coupled plasma system with a radio-frequency (RF) of 40 kHz and a maximum of 200 W power from Diener electronic. The detection limit of the chamber is 0.2 mbar. The chamber was kept at a high vacuum state (< 0.2 mbar) for 5 minutes before the flow of desired gas to preclude any unwanted gas. Then the chamber was flushed with desired gas (N_2 and Ar) at 0.7 mbar for 1 minute and vacuumed up to 0.2 mbar for six rounds. The procedure guarantee that only desired atmosphere is filled in the chamber. For nitrogen plasma, mild (0.7 mbar/10W) and strong (0.7 mbar/16 W) conditions were used respectively to fabricate vacancy-free N-doped graphene and vacancy-rich N-doped graphene. Argon plasma with a pressure of 0.4 mbar and power of 14 W was adopted to prepare vacancy-rich graphene. In the RRDE measurements, we also treated bare GC disk electrodes with the same conditions, to see if the GC, instead of RRDE graphene, would contribute to the ORR reaction.

Graphene electrode preparation. Two types of graphene electrodes including fresh graphene and RRDE graphene were prepared. Fresh graphene electrode was prepared in the following steps: 1) A thin layer of degassed epoxy resin (purchased from GENTEC) was pasted on a glass slide; 2) Graphene (on Cu film) was glued to the epoxy with the air face and left Cu film facing out; 3) Then, the resin was cured (crosslinking the epoxy resin with curing agent form a solid resin layer), to make the cured-solid resin a stable substrate for single-layer graphene at ambient condition in electrochemical conditions (~12 hours). Afterward, the Cu film was etched by ammonium persulfate (APS, purchased from Sigma Aldrich), and rinsed with ultrapure water (18.2 MΩ, by a Milli-Q system) three times. We electrically connect the resin-fixed graphene electrode to a copper wire with silver epoxy (purchased from GENTEC) and cover any exposed

part of other than fresh graphene with soft replica rubber (Flexbar Machine Corp.). For the RRDE graphene electrode, plasma-treated graphene was transferred onto the surface of another layer of CVD graphene on Cu using a PMMA-assisted method[1] to form bilayer graphene. In detail, PMMA (6% in anisole) was spin-coated on the plasma-treated graphene on a Cu film at 4000 rpm for 1 minute and baked on a hotplate at 80 °C for 20 minutes to further evaporate anisole. Then PMMA-graphene-Cu was floated on the surface of a 0.5 M APS etchant in a Petri dish to etch away Cu film and this procedure usually takes around 30 minutes. Afterward, PMMA-graphene was floated on fresh ultrapure water for 7 turns and 5 minutes for each turn to remove any potential Cu ions and APS salt on the graphene. Then we obtained single-layer graphene carried by PMMA. The PMMA-graphene was transferred to another graphene/Cu film. After drying under ambient conditions, with the additional PMMA-graphene on graphene/Cu film, we obtained a PMMA-bilayer graphene-Cu film. After the etching away of Cu in the same manner mentioned above including the resin steps, we obtained a bilayer graphene carried by PMMA. Then we used a GC disk electrode to fish up the bilayer graphene in a Petri dish and then dried the PMMA-bilayer graphene-GC disk under ambient conditions. To remove the PMMA, we immersed the PMMA-bilayer graphene-GC disk in pure acetone solvent for 30 minutes, followed by a dropwise wash with fresh acetone, isopropanol, and ethanol.

Characterization. The Raman spectra were recorded with an alpha300 R-Confocal Raman Imaging made by WITEC with a laser wavelength of 532 nm. For each Raman measurement, CVD graphene was transferred to a SiO_2/Si wafer with a SiO_2 layer of 285 nm in thickness by PMMA-assisted method. All the spectra were recorded at ambient conditions.

X-ray photoelectron spectroscopy (XPS) was recorded on a ThermoScientific K-Alpha instrument equipped with a monochromatic X-ray source (Al K_α = 1486.6 eV). The spectra were fitted by CasaXPS software with Gaussian (70%)-Lorentzian (30%) (GL(30)) line shape and Shirley background. The XPS characterization was conducted with CVD graphene on Cu film after specific treatment to avoid any residue or contaminants during the transfer.

Electrochemical measurements. The electrochemical measurements were conducted in a custom-made three-electrode electrochemical cell under room temperature and ambient conditions. The assembly of the measurement is a conventional three-electrode system with the graphene electrode as the working electrode (and RRDE graphene in the RRDE measurements), a graphite rod as the counter electrode, and a reversible hydrogen electrode (RHE, Pt mesh in

H_2 saturated electrolyte operating at the same pH and connected to cell via a Luggin capillary) as a reference electrode. The collection efficiency of the RRDE was calculated as reported in our previous work[2] via the reversible redox reaction of potassium ferricyanide. In detail, in a 0.1 M sodium perchlorate purged with argon, 1 mM potassium ferricyanide (III) was reduced at the glassy carbon disk to Fe(II) which can be re-oxidized at the platinum ring. The reversible hydrogen electrode (RHE) was used as the reference electrode with a Luggin capillary that was only in contact with 0.1 M sodium perchlorate. Based on the currents, the collection efficiency ratio of the ring was determined as 22.5%.

The glassware (the cell, glass connectors, glass adaptors, and stoppers) was thoroughly cleaned before use, by immersing the glassware in a 1 g/l $KMnO_4$ solution in 0.5 M H_2SO_4 (Sigma, reagent grade) overnight. Afterward, the glassware was rinsed 5–10 times with water. To re-oxidize any MnO_2 traces, water, a few drops of H_2O_2 (Merck Emprove, 35%), and H_2SO_4 (Merck) were added. Finally, the glassware was rinsed 5-10 times and boiled in water for a total of three times. Before any measurements, the glassware was cleaned by boiling in and copiously rinsing the glassware with water prior to each experiment.

Prior to the ORR measurements, argon was purged for at least 30 minutes to fully remove dissolved O_2 from the solution, followed by several CV scans to determine the capacitive current. The electrolyte was then purged with O_2 for 30 minutes, followed by more than 10 CV scans at a scan rate of 100 mV s^{-1} to assess the stability of the ORR current. The LSV results were obtained afterwards. For the LSV measurements, electrodes were cleaned in the following steps. For the fresh graphene electrode, after the removal of PMMA, the entire electrode was cleaned with fresh acetone, isopropanol, and ethanol. To avoid damage before measurements, we use cleaned Petri dishes and tweezers by boiling them in ultrapure water for 30 mins and flushing them with fresh ultrapure water before use. To connect the Cu wire to the graphene electrode, we solidified the Cu wire to graphene with silver epoxy (solidified under ambient conditions for 8h). For Pt parts in the reference electrode, they were regularly cleaned by flame. The graphite electrode is used in the research of graphene electrodes to prevent any metal contamination. We cleaned the graphite electrode before any measurements by sonication in water for 5 minutes to remove any potential contaminants from the electrolyte.

To avoid Pt contamination from the Pt ring to the GC disk, the ring electrode and GC electrode were polished separately. The GC electrode encapsulated in PEEK (polyether ether ketone) holder was polished before each measurement (with 1.0, 0.3, and 0.05 micron alumina slurry

(Buehler) on MicroCloth (Buehler) polishing cloths for 2 minutes followed by rinsing and sonicating the electrode in ultrapure water for 10 minutes). Then the polished GC disk was disassembled from the holder for the transfer of the graphene electrode. The ring electrode was polished with the RRDE holder and a PTFE insertion in the hole of the disk electrode, by the same procedure mentioned above. After the transfer of bilayer graphene on the GC disk, the RRDE graphene was inserted into the holder without any further operations to prevent damaging the graphene. The electrolyte solution, 0.1M NaOH, was purged using argon prior to each experiment for at least 30 minutes and the cell was kept under a flow of argon during the experiment. Then we conducted blank LSV to see if other peaks arise before ORR. For ORR, the electrolyte was purged with O_2 for at least 10 minutes prior to the measurement and was purged continuously during both LSC and RRDE measurements. All the potentials mentioned in this work are with respect to RHE. Potentiostats used here were purchased from Metrohm (PGSTAT204 for polarization measurement and PGSTAT302 for RRDE measurement) under the NOVA operation software. The current density was normalized to the surface area of exposed graphene. Experiments were conducted in pre-purged by Ar following saturation of O_2.

2. Supplementary Raman and XPS spectra

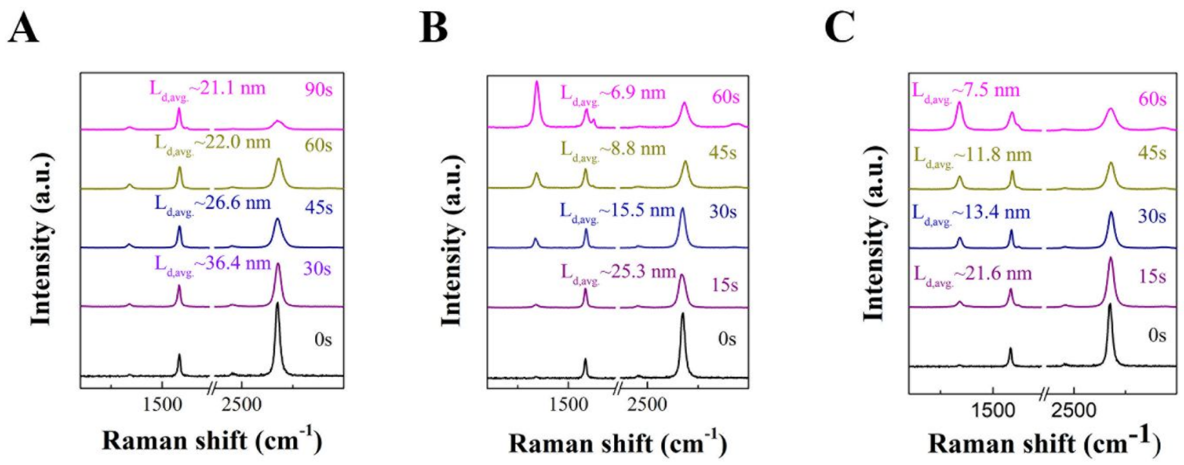

Figure S1. Raman spectrum of various exposure time. A, Raman spectra of VF-NGs. **B,** Raman spectra of V-NGs. **C,** Raman spectra of VGs.

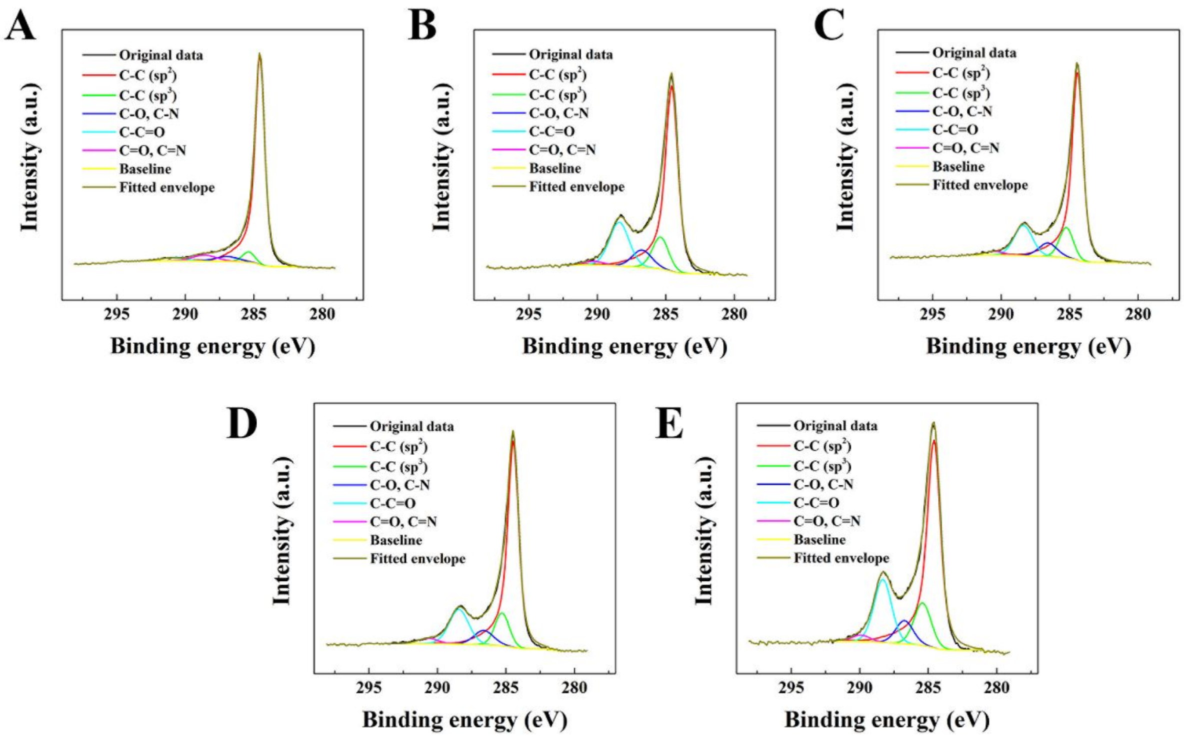

Figure S2. XPS C 1s spectrum. A, untreated graphene sample. **B,** VF-NG sample with a defect density of L_d ~ 36.4 nm. **C,** VF-NG sample with a defect density of L_d ~ 21.1 nm. **D,** V-NG sample with a defect density of L_d ~ 15.5 nm. **E,** V-NG sample with a defect density of L_d ~ 6.9 nm.

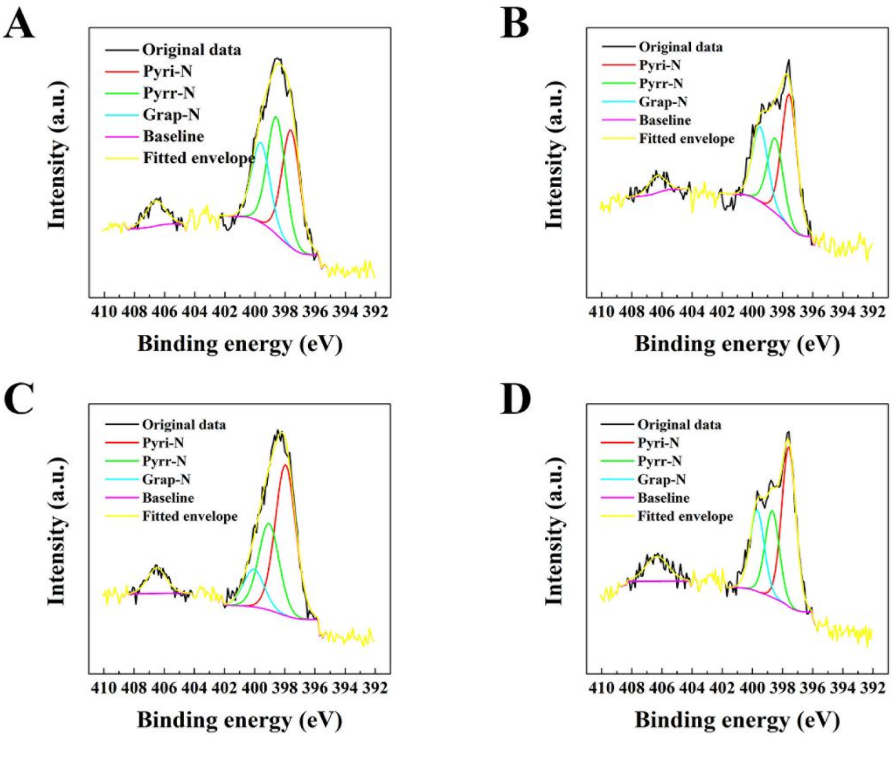

Figure S3. XPS N 1s spectrum. A, VF-NG sample with a defect density of L_d = 36.4 nm. **B,** VF-NG sample with a defect density of L_d = 21.1 nm. **C,** V-NG sample with a defect density of L_d = 15.5 nm. **D,** V-NG sample V-NG sample with a defect density of L_d = 6.9 nm.

3. Supplementary electrochemical results

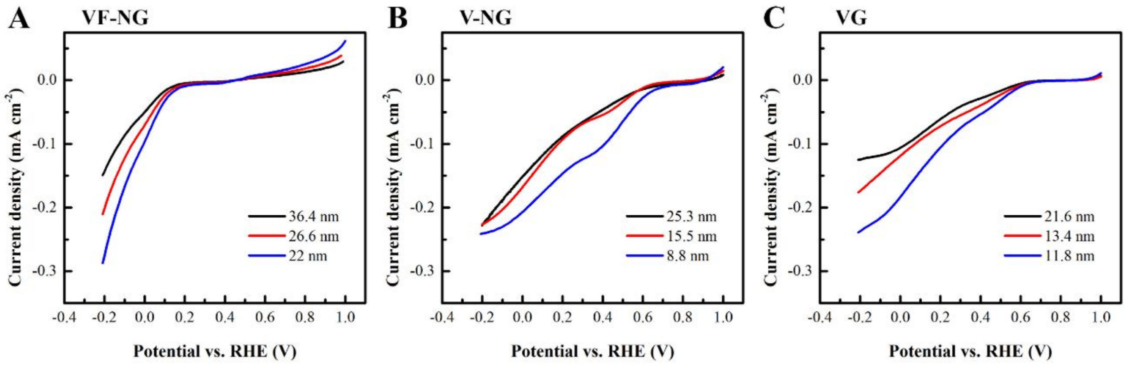

Figure S4. LSV curves of graphene with different defect densities. A, VF-NGs with L_d=36.4 nm, L_d=26.6 nm, and L_d=22 nm. **B,** V-NGs with L_d=25.3 nm, L_d=15.5 nm, and L_d=8.8 nm. **C,** VGs with L_d=21.6 nm, L_d=13.4 nm, and L_d=11.8 nm.

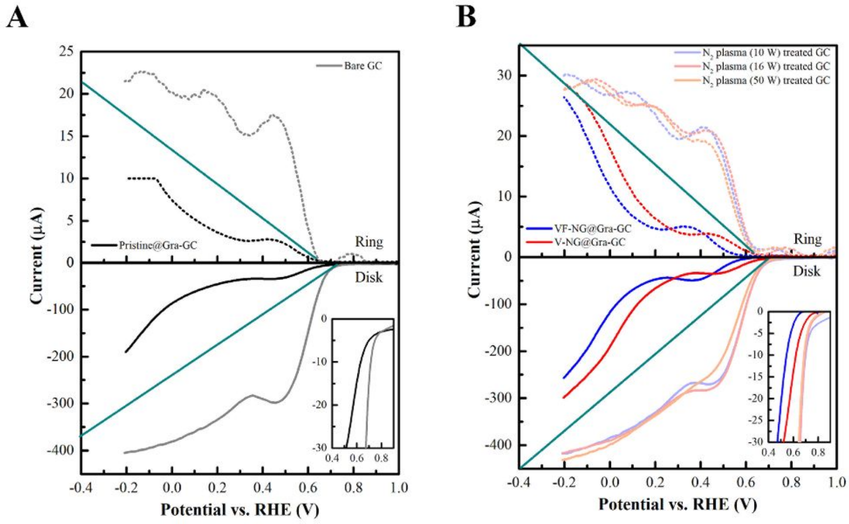

Figure S5. Ring-disk current plots of bare GC and Gra-GC. A. ring and disk currents of bare GC and RRDE with pristine graphene in 0.1M NaOH with 100 mV/s scan rate at 800 rpm. The large difference in both ring and disk currents between bare GC and graphene-covered GC indicates that the GC surface was fully covered by graphene. The measured currents for pristine RRDE graphene reflect the ORR that occurred at the graphene surface. Inset: zoom-in plots of the region close to the onset potential. **B.** Ring-disk currents of VF-NG and V-NG in

comparison with GC treated by a nitrogen plasma. The GCs were treated under 0.7 mbar N_2 atmosphere with 10 W (the same as VF-NG), 16 W (the same as V-NG), and 50 W. Inset: zoom-in plots of the region close to the onset potential.

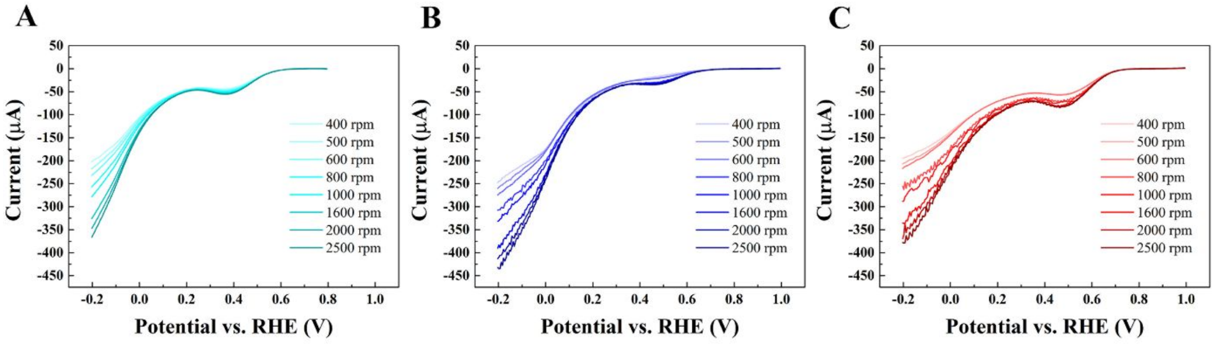

Figure S6. Disk currents at different rotation speeds. A, VF-NG. **B,** V-NG. **C,** VG.

A Koutecky-Levich (K-L) analysis was performed to compare the activity difference between nitrogen species and vacancy-containing graphene. As the following equation shows,

$$1/I_{disk} = 1/I_K + 1/I_L = 1/I_K + 1/(0.62 n F A D_O^{2/3} C_O v^{-1/6} \omega^{1/2})$$

where I_{disk} is the current measured from the disk, I_K is the kinetic current, I_L is the diffusion-limited current that can be obtained when the reaction rate is much larger than the mass transport rate, n is the electron transfer number, F is the Faraday constant (C mol^{-1}), A is the electrode area (cm^2), D_O is the diffusion coefficient of O_2 (cm^2 s^{-1}), C_O is the concentration of O_2 in the electrolyte, v is the kinematic viscosity (cm^2 s^{-1}) and ω the rotation rate (rad s^{-1}) .[3] Accordingly, the inverse of disk current at 0 V and -0.2 V was plotted as a function of the inverse square root of the rotation rates in Figure 4D. When the rotation rate reaches an infinite speed, the disk current would be no longer limited by mass transport, giving rise to the zero $1/I_L$ and the intercept at the y axis as $I_{disk} = I_K$. In other words, the non-zero intercept (namely I_K) suggests the kinetic limit for ORR at the graphene samples being recorded. A $1/I_k$ closer to zero suggests a higher kinetic current. Figure S7B presents the intercepts of K-L plots extracted from Figure S7A. The $1/I_K$ of VF-NG, V-NG, and VG is -5.3, -3.0, and -2.5 mA^{-1} at 0 V, suggesting VG is the most active graphene electrode among the studied samples. At a more negative voltage (-0.2 V), The $1/I_k$ of VF-NG, V-NG, and VG is -1.2, -1.1, and -0.8 mA^{-1}, further supporting the conclusion that VG is more active than VF-NG. Upon the same doping levels of vacancy and nitrogen species, VG (dominated by vacancy defects) contributes to the highest kinetic current with a higher selectivity toward water production while VF-NG (dominated by

nitrogen species) has the lowest activity with a higher selectivity toward hydrogen peroxide production.

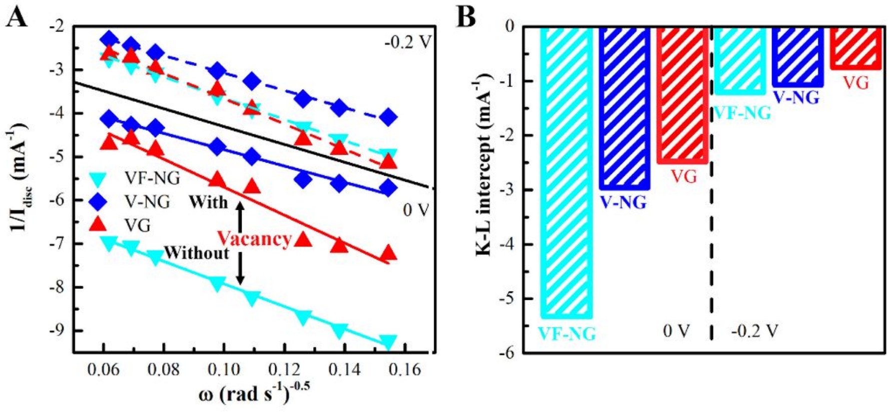

Figure S7. A, Koutecky-Levich (K-L) points with fitted lines of the inverse currents at 0 V (bottom, solid line) and -0.2 V (top, dash line) vs. RHE for different samples as a function of the inverse square root of the rotation rates. **B,** K-L intercepts extracted from **A** by linear fitting.

References

(1) Qing, F. Z.; Zhang, Y. F.; Niu, Y. T.; Stehle, R.; Chen, Y. F.; Li, X. S. Towards large-scale graphene transfer. *Nanoscale* **2020**, *12* (20), 10890-10911. DOI: 10.1039/d0nr01198c.

(2) Jiang, L.; van Dijk, B.; Wu, L. F.; Maheu, C.; Hofmann, J. P.; Tudor, V.; Koper, M. T. M.; Hetterscheid, D. G. H.; Schneider, G. F. Predoped Oxygenated Defects Activate Nitrogen-Doped Graphene for the Oxygen Reduction Reaction. *Acs Catalysis* **2022**, *12* (1), 173-182. DOI: 10.1021/acscatal.1c03662.

(3) Xu, S. C.; Kim, Y.; Higgins, D.; Yusuf, M.; Jaramillo, T. F.; Prinz, F. B. Building upon the Koutecky-Levich Equation for Evaluation of Next-Generation Oxygen Reduction Reaction Catalysts. *Electrochimica Acta* **2017**, *255*, 99-108. DOI: 10.1016/j.electacta.2017.09.145.
